# Examining How Preschool Teachers’ Positive Psychological Capital Impacts Digital Education Innovation: A Moderated Moderation Analysis of Effort Expectancy and Behavioral Intention

**DOI:** 10.3390/bs15070952

**Published:** 2025-07-14

**Authors:** Myoung-Sun Sung, Young-Eun Lee

**Affiliations:** Department of Early Childhood Education, College of Social Science, Gachon University, Seongnam-si 13120, Gyeonggi-do, Republic of Korea

**Keywords:** digital education, innovation behavior, positive psychological capital, preschool teachers, behavioral intention, effort expectancy

## Abstract

This study examines the impact of preschool teachers’ positive psychological capital on their digital education innovation behavior, focusing on the moderated moderation effects of digital education effort expectancy and behavioral intention on this relationship. Data were analyzed from 211 preschool teachers of children aged 3–5 years in South Korea. SPSS (version 25.0) was used to conduct descriptive and Pearson correlation analyses, and PROCESS Macro (version 4.3) was used to perform the moderation analysis. The results indicate that preschool teachers with higher positive psychological capital exhibited increased innovation behavior toward digital education, and this effect was further strengthened by higher effort expectancy. These research findings can provide useful foundational data for designing teacher training programs to promote preschool teachers’ digital education innovation behavior.

## 1. Introduction

Innovation behavior generates valuable and novel ideas in work settings, leading to improved work processes and enhanced performance (Jafri, 2010). Recently, generative artificial intelligence technologies, which create new content in forms such as text, images, and audio, have gained significant attention in the field of education. In this context, preschool teachers’ digital education innovation behavior, including the use of generative AI, is considered crucial for the effective implementation of digital education courses (Zhai, 2024). In South Korea, the Ministry of Education enacted legislation in 2022 regarding digital education in elementary, middle, and high schools. This law aims to establish a future-oriented smart educational environment, promote the sustainable informatization of education, provide personalized educational services through information and communication technology (ICT), and construct a shared digital infrastructure for educational information. Since 2023, various on-site support initiatives—such as digital play environments, digital competency enhancement, media literacy education, and AI—have been introduced for early childhood digital education (Ministry of Education, 2019). The above context indicates that preschool teachers, who provide education and programs for young children, need systematic support to enhance their digital education capabilities.

Positive psychological capital (PsyCap) refers to a positive mindset in which individuals believe they can achieve their goals (Stajkovic, 2006). Among preschool teachers, positive PsyCap contributes to positive emotions and improves their quality of life and job performance, including job crafting (Battistelli et al., 2023; Cheon & Lee, 2023). Özsungur (2019) discovered that people with positive PsyCap exert the necessary effort to complete difficult tasks, find new ways to achieve their goals, and demonstrate assertive behavior. Furthermore, positive PsyCap reduces workplace silence, enables individuals to actively engage in their work, and enhances their innovation behavior based on their motivation (Avey et al., 2011; Sweetman et al., 2011).

Perceived effort expectancy of digital education refers to “the degree to which a person believes that information systems are easy to use” (Venkatesh et al., 2003, p. 450). Thusi and Maduku (2020) analyzed the determinants of mobile banking app usage among South African millennials and found that consumers’ perceived ease of use of digital applications—that is, the belief that the technology is easy to use—significantly influenced their usage behavior. Additionally, in the field of management, effort expectancy has been proposed as a key factor in predicting innovation behavior, with research suggesting that higher effort expectancy leads to increased innovation behavior (Venkatesh et al., 2012). Thus, preschool teachers may exhibit digital educational innovation behavior when convinced that digital technologies are easy to learn and use.

Behavioral intention to adopt digital education is defined as “the degree of intention or plan to utilize an information system” (Venkatesh et al., 2003, p. 451). Davis (1989), who studied American information technology users, argued that the behavioral intention to use a system is a key determinant of actual system use, which is in line with the fact that preschool teachers’ behavioral intention to adopt digital education is closely linked to actual implementation. Su and Yang (2024) recommended establishing digital education policies to strengthen digital competence in early childhood education and consider the behavioral intentions of individual teachers to implement digital education effectively. Therefore, this study aims to examine the moderating effect of digital education effort expectancy on the relationship between preschool teachers’ positive PsyCap and digital education innovation behavior, and the moderated moderation effect of behavioral intention to adopt digital education on this interaction.

## 2. Literature Review and Theoretical Hypotheses

### 2.1. Digital Education Innovation Behaviors and Positive Psychological Capital

Researchers’ views on what constitutes innovation behavior vary. Scott and Bruce (1994) categorized innovation behavior into two stages, innovation recognition and implementation, whereas Janssen (2000) proposed three components—idea development, idea promotion, and idea implementation. In this study, we defined preschool teachers’ digital education innovation behavior as intentionally generating, promoting, and applying ideas related to digital technologies in the context of implementing teaching–learning methods.

Luthans and Youssef (2004) systematized positive psychology and viewed it as a form of organizational capital. Through a meta-analysis, they classified factors directly related to organizational performance into self-efficacy, hope, optimism, and resilience. Cheon and Lee (2023) further extended this framework by including extraversion and trust, which are important in fostering preschool teachers’ innovation behavior and collaboration environments. Therefore, this study defines positive PsyCap as an individual’s malleable positive psychological state, including self-efficacy, optimism, trust, extraversion, resilience, and hope.

South Korea’s Nuri Curriculum is a national-level preschool curriculum for all children ages 3 to 5 in South Korea (Kim, 2023). In Korea, national curricula such as the Nuri Curriculum emphasize the importance of preschool teachers’ digital education innovation behavior. However, research exploring the impact of teachers’ positive psycap on digital education innovation behavior remains limited. According to international studies, high levels of positive psycap promote motivation, agility, and professional engagement, and are predictive of innovation behavior (Wahid & Ayub, 2024; Yeo et al., 2022). Based on this, the first hypothesis is as follows.

**H1.**  *Higher positive PsyCap would increase preschool teachers’ innovation behavior toward digital education.*

### 2.2. The Moderating Effect of Digital Education Effort Expectancy

Effective teaching and learning can be achieved if preschool teachers believe that they can easily utilize digital technologies in ways appropriate for early childhood development. Venkatesh et al. (2012) proposed the Unified Theory of Acceptance and Use of Technology (UTAUT2), which found that four key factors, namely, performance expectancy, effort expectancy, social influence, and facilitating conditions, were useful in predicting users’ intention to adopt technology and usage behavior. In particular, effort expectancy is an important factor in perceiving the ease of using technology, which directly affects the intention to adopt technology. In a study conducted on university professors at Jos Plateau State University, Nigeria, Oye et al. (2011) used the UTAUT2 model to investigate the factors that influenced the acceptance and use of ICT by university professors, with effort expectancy emerging as the most significant predictor among the variables examined. Therefore, in this study, we defined digital education effort expectancy as the extent to which preschool teachers believe they can easily use digital education teaching and learning methods in their teaching practice. Based on this, the following second hypothesis is proposed.

**H2.**  *Digital education effort expectancy would moderate the relationship between preschool teachers’ positive PsyCap and digital education innovation behaviors. When preschool teachers’ positive PsyCap is high, digital education innovation behaviors would increase, and preschool teachers’ digital education effort expectancy would strengthen this relationship.*

### 2.3. Moderators of Behavioral Intention to Adopt Digital Education

The use of digital technology in early childhood education has long been limited (Taulany & Fauziah, 2018). This is because a protection-oriented approach has been prioritized over the use of technology, based on concerns that young children’s exposure to digital media could result in negative outcomes such as language development delays, social isolation, and addiction (Finch & Arrow, 2017). However, with the rapid proliferation of smart devices and generative AI across classrooms, homes, and play environments, it is no longer a realistic response to completely shield young children from digital environments (Hannaway, 2024). Accordingly, there is a growing need for an educational shift toward helping young children learn safely and meaningfully within digital environments.

According to recent studies, young children can effectively develop a range of cognitive and non-cognitive skills—such as computational thinking, collaboration, and problem solving—through age-appropriate digital education (Possekel, 2023). In addition, Zhai (2024) focused on the varying roles of pre-service and in-service early childhood educators in the United States in their use of generative artificial intelligence (GenAI), identifying a four-stage framework: Observer, Adopter, Collaborator, and Innovator. This framework outlines progression from mere acceptance to full innovation. To ensure the stable implementation of these positive outcomes in early childhood education settings, preschool teachers’ behavioral intention to adopt digital education plays a critical role. For instance, Yang and Park (2022) reported that preschool teachers’ knowledge of AI-related content and instructional experience positively influence their behavioral intention to accept AI education. Furthermore, Chen (2017), in a study involving preschool teachers in Taiwan, identified a structural pathway in which behavioral intention to use digital learning tools significantly predicted innovation behavior in instructional design. The findings highlight that in the context of digital education in early childhood settings, teachers’ behavioral intention to adopt digital technologies serves as a critical factor in promoting innovative pedagogical practices. Based on this, the current study defines behavioral intention to adopt digital education as the extent to which pre-school teachers intend or plan to utilize digital education in their teaching and learning activities. Accordingly, the following third hypothesis is proposed.

**H3.**  *Among preschool teachers, the positive effect of positive PsyCap on digital education innovation behavior is expected to be strengthened by effort expectancy, and this moderating effect is further amplified when behavioral intention to adopt digital education is high, resulting in a moderated moderation effect.*

**Figure 1:**
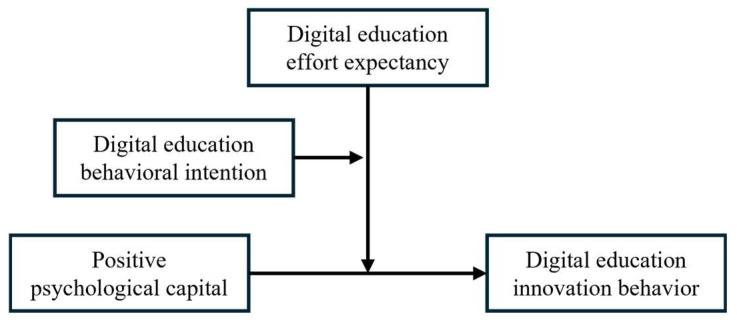
Moderated moderation effect model.

The moderated moderation effect model used in this study is presented in Figure 1.

## 3. Materials and Methods

### 3.1. Participants

Purposive sampling was used to select 231 preschool teachers from Gyeonggi Province, South Korea, and the questionnaire was written in Korean. After the data cleaning process, a total of 231 valid responses were obtained. To obtain more accurate results, those who responded with the same pattern check or outlier values were additionally excluded, and the final 211 valid responses were used in all subsequent analyses. Women represented 99.5% (*n* = 210) of the sample, and the age distribution was 48.3% (*n* = 102) in their 30s, 45.0% (*n* = 95) in their 20s, and 3.3% (*n* = 7) in their 40s and 50s. In terms of education, 58.8% (*n* = 124) had a college degree, 38.4% (*n* = 81) had a two-year college degree, and 2.8% (*n* = 6) had a graduate degree or higher.

### 3.2. Measures

Four scales were used in the study. The positive PsyCap scale was modified and supplemented to suit Korean preschool teachers (Cheon & Lee, 2023). The three scales of digital education innovation behavior, effort expectancy, and behavioral intention are scales developed in the field of business administration, and one professor of early childhood education and four directors of early childhood education in Korea conducted content validity verification. The content validity evaluation results showed that the three scales effectively measured factors related to the digital education of Korean preschool teachers. All four scales are 5-point Likert scales from 1 (not at all) to 5 (very much so).

#### 3.2.1. Digital Education Innovation Behavior Scale

Digital education innovation behavior was measured using the innovative work behavior scale of the multidimensional perspective (IWB) developed by Janssen (2000) following Scott and Bruce (1994). This scale was modified and supplemented to suit Korean preschool teachers, consists of three subfactors, idea generation, idea promotion, and idea realization, and consists of a total of nine items. Example items are “idea generation: I develop new ideas to solve difficult problems related to digital education (digital, media, AI literacy education, AI utilization education, AI value education)”, “idea catalysis: I try to gain support for new digital education (digital, media, AI literacy education, AI utilization education, AI value education) ideas that are different from what has been done before”, and “idea realization: I refine new digital education (digital, media, AI literacy education, AI utilization education, AI value education) ideas to make them useful”. The subfactors were averaged, and a higher average value indicated higher digital education innovation behavior. Cronbach’s α coefficient was 0.92.

#### 3.2.2. Positive Psychological Capital Scale

Based on Tösten and Özgan’s (2014) scale, Cheon and Lee (2023) developed a modified scale for Korean preschool teachers. It consists of six subscales (self-efficacy, optimism, trust, extraversion, resilience, and hope) with a total of 26 items. Sample items include “self-efficacy: I am confident that I can do my job well”, “optimism: I have a lot of energy”, “trust: I am aware of my professional responsibilities as a teacher”, “extraversion: I can share information about my job in groups related to my profession”, “resilience: I can address negative environmental factors to improve the education of young children”, and “hope: I grow as a person by experiencing difficult things”. The subscales were averaged, with higher mean values indicating more positive PsyCap. Cronbach’s α coefficient was 0.96.

#### 3.2.3. Digital Education Effort Expectancy Scale

In this study, the effort expectancy scale developed by Yang and Park (2022) was used. This scale is an adaptation of a scale based on the Unified Theory of Acceptance and Use of Technology (UTAUT2) by Venkatesh et al. (2012). The scale consists of four items, and an example question is “Learning to teach and learn about digital education (digital, media, AI literacy, AI utilization, and AI value education) is easy for me”. Higher mean values indicate higher digital education effort expectancies. Cronbach’s α coefficient was 0.81.

#### 3.2.4. Digital Education Behavioral Intention Scale

The digital education behavioral intention scale was adapted from Venkatesh et al. (2012) and modified and supplemented by Yang and Park (2022). This scale was modified and supplemented to suit Korean preschool teachers. The scale consists of five items, and an example question is “I am willing to use digital education (digital, media, AI literacy, AI utilization, and AI value education) in early childhood education”. A higher mean value indicates a higher behavioral intention to adopt digital education. Cronbach’s α coefficient was 0.70.

### 3.3. Analytical Approach

The SPSS 25.0 program was used in this study to analyze the data collected. Cronbach’s α was used to measure the reliability of each variable. Frequency analysis was conducted to examine the respondents’ characteristics such as gender, age, education level, institution, position, classroom, and years of service. Additionally, to test the moderating effects of the main variables while controlling for the educational level of preschool teachers, a moderation analysis was conducted using Model 3 in Process Macro version 4.3 (Hayes, 2017). To eliminate the multicollinearity problem of the interaction term between the independent variable and the moderating variable, the variables were mean-centered and used in the analysis. The regression models adopted in this study are listed as follows. In this regression model, ‘α’ represents the basic level of digital education innovation behavior when all the influencing factors, namely, positive PsyCap, effort expectancy for digital education, and behavioral intention, are all 0. In other words, it can be said to be the initial value of digital education innovation behavior that appears when there is no influence. Meanwhile, the ‘b’ coefficient shows how each variable or the interaction between variables affects digital education innovation behavior.

$$\begin{array}{cc} \mathrm{Performance}=\alpha 1 & +\;b1\mathrm{Positive}\;\mathrm{PsyCap}\\ & +\;b2\mathrm{Digital}\;\mathrm{education}\;\mathrm{effort}\;\mathrm{expectancy}\\ & +\;b3\mathrm{Behavioral}\;\mathrm{intention}\;\mathrm{toward}\;\mathrm{digital}\;\mathrm{education}\\ & +\;b4\;(\mathrm{Positive}\;\mathrm{PsyCap}\times \mathrm{Digital}\;\mathrm{education}\;\mathrm{effort}\;\mathrm{expectancy})\\ & +\;b5\;(\mathrm{Positive}\;\mathrm{PsyCap}\times \mathrm{Digital}\;\mathrm{education}\;\mathrm{behavioral}\;\mathrm{intention})\\ & +\;b6\;(\mathrm{Digital}\;\mathrm{education}\;\mathrm{effort}\;\mathrm{expectancy}\times \mathrm{Digital}\;\mathrm{education}\;\mathrm{behavioral}\;\mathrm{intention})\\ & +\;b7\;(\mathrm{Positive}\;\mathrm{PsyCap}\times \mathrm{Digital}\;\mathrm{education}\;\mathrm{effort}\;\mathrm{expectancy}\times \mathrm{Digital}\;\mathrm{education}\;\mathrm{behavioral}\;\mathrm{intention}) \end{array}$$

## 4. Results

### 4.1. Descriptive Statistics and Correlation Analysis

Table 1 presents the results of the correlation analyses of the main variables. The correlation coefficients (r) among the independent variables were less than 0.8, indicating that none of the variables are suspected of multicollinearity according to Field’s (2013) criteria. In addition, consistent with Kline’s (2016) criteria, the minimum and maximum values were within the normal range, skewness was below the absolute value of three, and kurtosis was below the absolute value of 10, thus satisfying the assumption of normality.

### 4.2. Moderated Moderation Effects

The moderated moderation analysis was conducted using Model 3 of Hayes (2017) PROCESS macro. Table 2 presents the results of this study. The independent variable, positive PsyCap (β = 0.59, *p* < 0.001), had a positive effect on the dependent variable, digital education innovation behavior. This finding supports Hypothesis 1. In addition, the first moderator variable, effort expectancy in digital education, had a positive moderating effect on the relationship between positive psycap and digital education innovation behavior (β = 0.36, *p* = 0.005). This finding supports Hypothesis 2. Finally, the moderating effect of preschool teachers’ positive psycap on the relationship between digital education innovation behaviors and effort expectancy was a positive moderated moderation effect of behavioral intention to adopt digital education (β = 0.30, *p* = 0.013).

According to Hayes (2017), moderated moderation interaction effects can be interpreted as eight different types of three-way interaction effects, depending on the coefficient of the effect of the independent variable (X) on the dependent variable (Y), the coefficient of the interaction between the independent variable (X) and the first moderator (W), the combination of the coefficient of the interaction between the independent variable (X) and the first moderator (W), and the coefficient of the interaction between the independent variable (X) and the second moderator (Z). In this study, as the independent variable (X) increased, the dependent variable (Y) also increased, and when the first moderating variable (W) increased, the positive effect of the independent variable (X) on the dependent variable (Y) became stronger. This two-way interaction is strengthened when the second moderating variable (Z) increases (Hayes, 2017). Applying this, we found that as preschool teachers’ positive psycap increases, their digital education innovation behavior also increases (β = 0.59, *p* < 0.001), and the positive effect of positive PsyCap on digital education innovation behavior becomes stronger when the first moderator, effort expectancy for digital education, increases (β = 0.36, *p* = 0.005). This two-way interaction effect is further strengthened when the second moderator, behavioral intention to adopt digital education, increases (β = 0.30, *p* = 0.013). Hence, Hypothesis 3 is supported. This moderated moderating effect had an explanatory power of 1% and was significant at the 0.01 level.

### 4.3. Significance Testing and Graphing of Interactions by Condition

The researchers tested the significance of the interaction between digital education behavioral intention and the moderating variable level of digital education effort expectancy. Following Aiken et al. (1991), we defined the High Group as having one standard deviation (+SD) higher than the mean (*M*) and the Low Group as having one standard deviation (−SD) lower than the mean (*M*) and then tested the moderating effect.

The results of the moderation analysis examining the impact of positive psycap on digital education innovation behavior according to the level of effort expectancy toward digital education (high, average, low) are as follows. Among preschool teachers with high effort expectancy, positive psycap had a significant positive effect on digital education innovation behavior across all levels of behavioral intention (high: *t* = 5.34, *p* < 0.001, 95% CI [0.56, 1.23]; average: *t* = 7.00, *p* < 0.001, 95% CI [0.62, 1.11]; low: *t* = 8.39, *p* < 0.001, 95% CI [0.64, 1.03]). Similarly, among those with average effort expectancy, positive psycap also had a significant positive effect on digital education innovation behavior at all levels of behavioral intention (high: *t* = 4.43, *p* < 0.001, 95% CI [0.29, 0.73]; average: *t* = 7.21, *p* < 0.001, 95% CI [0.43, 0.76]; low: *t* = 10.14, *p* < 0.001, 95% CI [0.55, 0.81]). On the other hand, among preschool teachers with low effort expectancy toward digital education, positive psycap had a significant effect on digital education innovation behavior only in groups with average behavioral intention (*t* = 2.51, *p* = 0.013, 95% CI [0.07, 0.58]) and low behavioral intention (*t* = 5.78, *p* < 0.001, 95% CI [0.35, 0.71]). No statistically significant effect was observed in the group with high behavioral intention.

**Figure 2:**
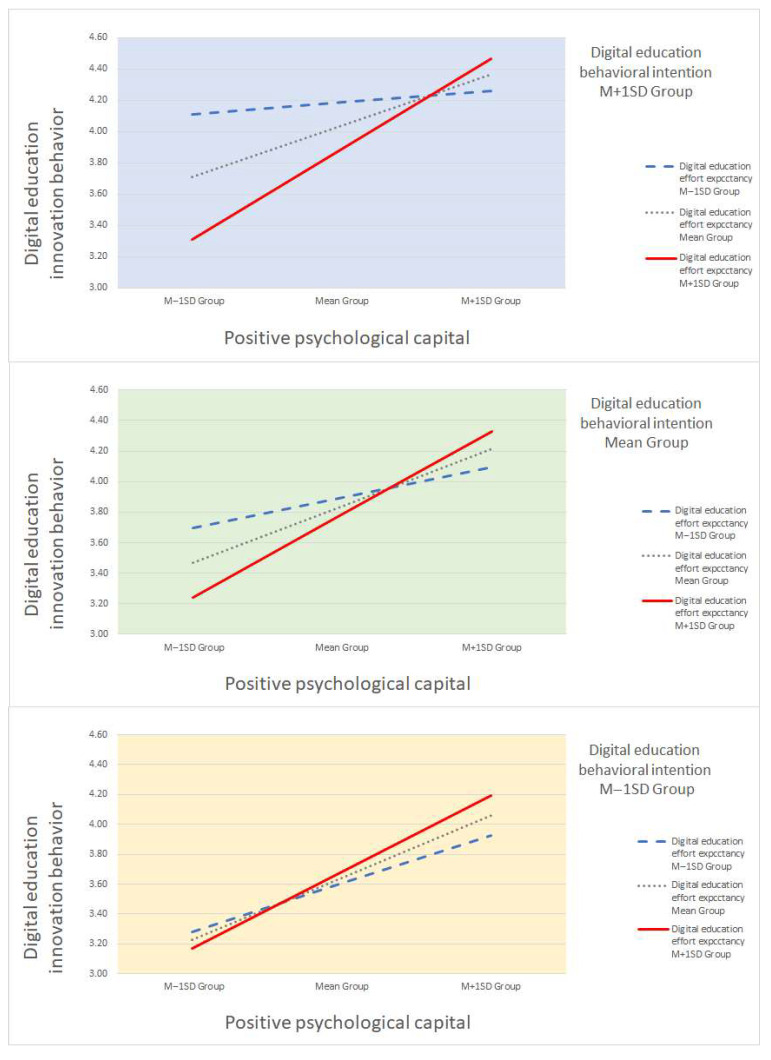
Moderated moderation effect. Note. M, mean; SD, standard deviation.

The results indicate that the effect of positive PsyCap on digital education innovation behavior depends on the level of digital education effort expectancy and behavioral intention. Specifically, the extent to which positive PsyCap increases digital education innovation behavior is greater when both effort expectancy and behavioral intention are high, as illustrated by the steepest slope in Figure 2. Conversely, when behavioral intention is high but effort expectancy is low, the effect of positive PsyCap is minimal, as indicated by the flat slope.

## 5. Discussion

### 5.1. Impact of Positive Psychological Capital on Digital Education Innovation Behavior

The results support Hypothesis 1, confirming that positive PsyCap significantly enhances digital education innovation behavior among preschool teachers. Although few prior studies have specifically targeted early childhood educators in digital contexts, findings from other domains support a consistent relationship between positive PsyCap and innovation behavior.

For example, Khoshaim (2024) examined university students in e-learning settings and found that students with higher positive PsyCap demonstrated greater digital engagement and academic innovation. Lee (2016), studying Korean university students in accounting information systems courses, reported that positive PsyCap enhanced engagement with AI-based learning content. In the corporate domain, Dimas et al. (2022) found that employees in Portugal showed improved team innovation when team-level positive PsyCap was high, mediated by team learning. In the field of information technology, Ziyae et al. (2015) reported that among banking employees in Iran, the resilience component of positive PsyCap was particularly crucial for fostering innovation in uncertain environments.

These diverse findings suggest a cross-contextual function of positive PsyCap as a psychological resource that enables individuals to respond proactively in situations requiring adaptation and creativity. Applied to early childhood education, this implies that positive PsyCap equips teachers with internal strength to confront and embrace digital change, especially in complex or unfamiliar environments involving emerging technologies.

### 5.2. Moderating Effect of Effort Expectancy on the Positive PsyCap–Innovation Relationship

The results support Hypothesis 2 by confirming that digital education effort expectancy significantly moderates the relationship between positive PsyCap and innovation behavior. That is, the positive influence of positive PsyCap on innovation behavior is stronger when preschool teachers perceive digital education tools as easy to use.

Although this study focuses on early childhood educators, the moderating role of effort expectancy has been widely supported in other settings. For example, Duong et al. (2023) reported that among Vietnamese university students, perceived ease of use of ChatGPT significantly enhanced their behavioral intention and actual use behavior. Similarly, Setiawan et al. (2024) found that Indonesian college students with higher self-efficacy had higher effort expectancy, which in turn promoted digital tool adoption.

These studies—though focused on higher education—align with the current findings in demonstrating that psychological readiness alone is insufficient; individuals must also perceive the task as manageable to initiate innovative behavior. In the context of early childhood education, this suggests that teachers may have the motivation and psychological resources (positive PsyCap) but still refrain from digital innovation if they perceive technological tools as difficult or burdensome. Thus, enhancing effort expectancy is essential for fully activating the innovation potential of teachers with high positive PsyCap.

### 5.3. Moderated Moderation: Behavioral Intention as a Catalytic Factor

The findings of this study support Hypothesis 3, revealing a significant three-way interaction among positive PsyCap, digital education effort expectancy, and behavioral intention to adopt digital education. Specifically, the effect of positive PsyCap on innovation behavior was most pronounced when both effort expectancy and behavioral intention were high. This indicates that behavioral intention acts as a key motivational amplifier that enables psychological and cognitive resources to translate more effectively into innovation behavior.

As shown in Figure 2, the strength of this relationship varied by the level of behavioral intention. When behavioral intention was high, the relationship between positive PsyCap and innovation behavior became considerably stronger as effort expectancy increased, as evidenced by the steep slope in the graph. This suggests that preschool teachers who not only possess strong psychological resources and perceive digital tools as easy to use but also hold clear intentions to implement such tools are more likely to demonstrate innovative behaviors in digital education. By contrast, when behavioral intention was low, the slopes remained nearly flat regardless of the level of effort expectancy. This implies that even when teachers are psychologically prepared and find the technology accessible, innovation behavior may not manifest without sufficient intention. At moderate levels of behavioral intention, the effect of positive PsyCap on innovation behavior gradually increased with effort expectancy, indicating an intermediate amplification effect.

These findings suggest that behavioral intention functions not as a standalone antecedent to behavior but as a moderator that enhances the effect of positive PsyCap and effort expectancy on innovation. It acts as a psychological catalyst in situations where multiple conditions must be met for innovation to occur—specifically in the context of early childhood education, where digital transformation is still emerging.

This pattern partially aligns with the Technology Acceptance Model (TAM), which proposes that perceived ease of use leads to behavioral intention, which in turn predicts actual system usage (Davis, 1989). Supporting this framework, Pham et al. (2020) found that among university lecturers in Vietnam, perceived ease of using instructional technologies significantly increased their intention to adopt such tools. Similarly, Wang et al. (2024) reported that while Chinese preservice teachers exhibited strong intention to use generative AI in lesson planning, actual usage remained low when effort expectancy was limited.

However, unlike these traditional TAM-based models, the present study introduces a more complex moderated moderation framework that reflects the unique characteristics of early childhood education. In this model, effort expectancy does not directly influence behavioral intention or behavior but instead moderates the relationship between positive PsyCap and innovation behavior. Behavioral intention, in turn, moderates this moderation, enhancing or dampening the effect depending on its level.

Importantly, in early childhood education, teachers’ behavioral intention to adopt digital tools is not solely shaped by their perceptions of ease or usefulness, but also by more context-specific factors such as personal pedagogical values, institutional culture, and beliefs about developmentally appropriate practice. For example, Vidal-Hall et al. (2020) highlighted that early childhood educators’ willingness to use digital media is strongly influenced by their philosophical commitment to child-centered learning. Similarly, Redeș et al. (2023) found that the organizational climate and leadership support within preschools significantly affected teachers’ intention to engage in integrative, technology-enhanced teaching behaviors.

Thus, in contrast to TAM’s more linear formulation, this study suggests that behavioral intention is not merely an outcome of usability beliefs but is shaped by deeper contextual factors and, in turn, plays an enabling role in reinforcing the combined influence of positive PsyCap and effort expectancy. These findings highlight the importance of designing multifaceted interventions—ones that enhance not only teachers’ digital skills and psychological capital but also foster institutional conditions and pedagogical cultures that support intentional innovation in early childhood settings.

## 6. Conclusions and Limitations

### 6.1. Limitations

This study has several limitations. First, digital education innovation behavior was assessed through self-reported questionnaires completed by preschool teachers. While self-reports are widely used in behavioral research, they are inherently vulnerable to potential biases, including social desirability and selective recall. These limitations may affect the accuracy of responses, particularly when evaluating behaviors perceived as professionally desirable. Future research should complement self-report instruments with more objective and context-sensitive methods such as semi-structured interviews and field observations. Specifically, in situ data collection on teachers’ actual use of digital tools, their interaction patterns with children, and children’s engagement levels would help validate and enrich the findings.

Second, this study was conducted exclusively among South Korean preschool teachers. While this sample provides meaningful insight into a technologically advanced yet pedagogically child-centered national context, the findings may not be readily generalizable to other cultural or institutional environments. Differences in teacher training systems, national curriculum standards, and digital infrastructure may yield variation in how positive psycap, effort expectancy, and behavioral intention operate across settings. Future studies should explore these dynamics in more diverse educational and sociocultural contexts to examine the robustness and transferability of the proposed model.

### 6.2. Contributions

Despite these limitations, this study offers several important academic and practical contributions. First, it empirically investigates the psychological and cognitive mechanisms underlying preschool teachers’ digital education innovation behavior—an area that has received limited attention in early childhood education research. By introducing a moderated moderation framework, this study advances the understanding of how positive psychological capital interacts with effort expectancy and behavioral intention to influence innovation behavior in complex educational settings.

Second, while previous studies have largely adopted a linear Technology Acceptance Model (TAM) or UTAUT2 perspective, this study challenges and extends these models by demonstrating that behavioral intention may not only follow from perceived ease of use but also function as a higher-order moderator that enables or constrains the effect of other internal variables. This reconceptualization is particularly relevant in early childhood education, where intention is shaped by pedagogical beliefs, institutional culture, and perceptions of developmentally appropriate practice, as emphasized by Vidal-Hall et al. (2020) and Redeș et al. (2023).

Third, from a policy and training perspective, the findings highlight the need for multidimensional interventions. It is not sufficient to provide technical training alone. Supporting preschool teachers’ digital innovation requires fostering their positive psycap (e.g., self-efficacy, optimism, resilience), improving their perceptions of digital tool usability (effort expectancy), and cultivating strong behavioral intentions rooted in meaningful pedagogical goals. These insights can inform the design of teacher education programs, leadership development, and national digital education strategies that are developmentally aligned and contextually sensitive.

## Figures and Tables

**Table 1:** Descriptive statistics and correlations.

Variable	Min	Max	Mean	*SD*	Skewness	Kurtosis	1	2	3	4	5
1. Positive psychological capital	2.00	5.00	4.03	0.67	−1.40	1.54	--				
2. Digital education innovation behavior	1.89	5.00	3.80	0.71	−1.11	0.93	0.79 ***	--			
3. Digital education effort expectancy	2.00	5.00	3.90	0.75	−0.87	0.28	0.09	0.10	--		
4. Digital education behavioral intention	2.80	5.00	4.08	0.53	−0.52	−0.66	0.78 ***	0.72 ***	0.08	--	
5. Educational level	2.00	4.00	2.64	0.54	−0.04	−0.90	−0.09	0.00	−0.12	−0.01	--

Note. *N* = 211; *** *p* < 0.001; 1: positive psychological capital; 2: digital education innovation behavior; 3: digital education effort expectancy; 4: digital education behavioral intention; and 5: education (entered as a control).

**Table 2:** Moderated moderating effect.

Model	β	*SE*	*t*	*p*	95% CI [LL, UL]
*α*: Constant	3.73	0.05	78.05 ***	<0.001	[3.64, 3.83]
b1: Positive psychological capital	0.59	0.08	7.21 ***	<0.001	[0.43, 0.76]
b2: Digital education effort expectancy	−0.08	0.05	−1.60	0.112	[−0.19, 0.02]
b3: Digital education behavioral intention	0.34	0.09	4.05 ***	<0.001	[0.18, 0.51]
b4: Positive psychological capital × Digital education effort expectancy	0.36	0.13	2.81 **	0.005	[0.11, 0.61]
b5: Positive psychological capital × Digital education behavioral intention	−0.16	0.09	−1.92	0.056	[−0.33, 0.00]
b6: Digital education effort expectancy × Digital education behavioral intention	−0.29	0.13	−2.21	0.028	[−0.54, −0.03]
b7: Positive psychological capital × Digital education effort expectancy × Digital education behavioral intention	0.30	0.12	2.51 *	0.013	[0.07, 0.54]
Graduated from a 4-year university	0.22	0.06	3.84	<0.001	[0.11, 0.34]
Graduate school student or higher	−0.36	0.17	−2.14	0.034	[−0.70, −0.03]
*F* = 54.32 ***, *R*^2^ = 0.71, △*R*^2^ = 0.01 (*F* = 6.32 *)					

Note. *N* = 211; * *p* < 0.05; ** *p* < 0.01; *** *p* < 0.001; β, unstandardized regression coefficient; CI, confidence interval; LL, lower level; *p*, probability; SE, standard error; *t*, *t*-test statistic; UL, upper level; *F*, *F*-statistic from ANOVA; *R*^2^, coefficient of determination; △*R*^2^, change in the coefficient of determination.

## Data Availability

The data presented in this study are available on request from the corresponding author due to privacy.

## Abbreviations

The following abbreviations are used in this manuscript:

AI	Artificial intelligence;
ICT	Information and communication technology;
PsyCap	Psychological capital;
UTAUT2	Unified Theory of Acceptance and Use of Technology 2;
IWB	Innovative Work Behavior;
TAM	Technology Acceptance Model.
